# φBO1E, a newly discovered lytic bacteriophage targeting carbapenemase-producing *Klebsiella pneumoniae* of the pandemic Clonal Group 258 clade II lineage

**DOI:** 10.1038/s41598-017-02788-9

**Published:** 2017-06-01

**Authors:** Marco Maria D’Andrea, Pasquale Marmo, Lucia Henrici De Angelis, Mattia Palmieri, Nagaia Ciacci, Gustavo Di Lallo, Elisa Demattè, Elisa Vannuccini, Pietro Lupetti, Gian Maria Rossolini, Maria Cristina Thaller

**Affiliations:** 1 Department of Medical Biotechnologies, University of Siena, Siena, Italy; 2 Department of Experimental and Clinical Medicine, University of Florence, Florence, Italy; 3 Department of Biology, University of Rome “Tor Vergata”, Rome, Italy; 4 Department of Life Sciences, University of Siena, Siena, Italy; 5 Clinical Microbiology and Virology Unit, Florence Careggi University Hospital, Florence, Italy

## Abstract

The pandemic dissemination of KPC carbapenemase-producing *Klebsiella pneumoniae* (KPC-KP) represents a major public health problem, given their extensive multidrug resistance profiles and primary role in causing healthcare-associated infections. This phenomenon has largely been contributed by strains of Clonal Group (CG) 258, mostly of clade II, which in some areas represent the majority of KPC-KP isolates. Here we have characterized a newly discovered lytic *Podoviridae*, named φBO1E, targeting KPC-KP strains of clade II lineage of CG258. Genomic sequencing revealed that φBO1E belongs to the *Kp34virus* genus (87% nucleotide identity to vB_KpnP_SU552A). ΦBO1E was stable over a broad pH and temperature range, exhibited strict specificity for *K. pneumoniae* strains of clade II of CG258, and was unable to establish lysogeny. In a *Galleria mellonella* infection model, φBO1E was able to protect larvae from death following infection with KPC-KP strains of clade II of CG258, including one colistin resistant strain characterized by a hypermucoviscous phenotype. To our best knowledge φBO1E is the first characterized lytic phage targeting *K. pneumoniae* strains of this pandemic clonal lineage. As such, it could be of potential interest to develop new agents for treatment of KPC-KP infections and for decolonization of subjects chronically colonized by these resistant superbugs.

## Introduction

Resistance to antibiotics that for decades were successfully employed to treat bacterial infections has reached an alarming level, and we are increasingly challenged by multidrug resistant (MDR) or extremely drug resistant (XDR) pathogens for which very few effective therapeutic alternatives remain available^1, 2^. This phenomenon has become so widespread and worrisome that some international organizations forecast, for the 21^th^ century, the advent of a post-antibiotic era in which even common infections could be very difficult if not impossible to manage^3, 4^. In addition, as a result of globalization and increasing international mobility, antibiotic-resistant microorganisms tend to disseminate rapidly through different countries and, sometimes, evolve into real pandemics.

One of the most worrisome resistant pathogens undergoing pandemic dissemination is represented by *Klebsiella pneumoniae* producing KPC-type carbapenemases (KPC-KP), which very frequently show an MDR or even an XDR phenotype, including last resort molecules such as colistin. KPC-KP have spread rapidly and became endemic in several countries (e.g. USA, China, Taiwan, Israel, Greece, Italy and Colombia), where they are now a major cause of healthcare-associated infections correlated with high morbidity and mortality rates^5–10^.

The pandemic diffusion of KPC-KP was largely contributed by isolates belonging to few sequence types (STs), commonly ST11, which is the most prevalent KPC-KP detected in Asian countries (particularly in China and Taiwan)^11, 12^, and ST258 and variants thereof (e.g. ST512, ST554 and ST1879), collectively included in Clonal Group 258 (CG258), that experienced a wider dissemination^7^. Recent studies demonstrated that strains of ST258 are hybrid clones originated by a number of large genomic recombination events that gave rise to two major clades (clade I and clade II), characterized mainly by different capsule polysaccharide (CPS) gene clusters^13–15^. Clade II strains contribute to the majority of ST258 and of other STs of CG258 causing outbreaks reported in several regions including USA, Italy, South America, Israel and Greece^14, 16–20^.

Bacteriophages, which in the pre-antibiotic era were successfully used for the treatment of bacterial infections, were recently reevaluated to fight some infections, especially those sustained by MDR bacteria. The present work describes the isolation and characterization of a newly discovered lytic bacteriophage able to specifically lyse strains of *K. pneumoniae* of CG258 clade II, which is of potential interest to develop new bacteriophage-derived agents against KPC-KP.

## Results

### Isolation and preliminary characterization of phage φBO1E

Phage φBO1E was isolated in September 2013 from the wastewater system of a large tertiary-care teaching hospital located in central Italy, where KPC-KP have been endemic since 2011. Screening of untreated wastewaters for the presence of phages able to lyse the *K. pneumoniae* ST258 clade II test strain KKBO-1 resulted in several plaques that differed in size, all surrounded by a hazy halo zone, suggesting the ability by these phages to produce soluble, polysaccharide-degrading enzymes^21^. Three plaques, characterized by different morphology, were purified and amplified for further analysis, as described in the methods section. The restriction analysis of phage DNA obtained from the three selected preparations with HindIII and BamHI showed an identical profile, suggesting that the phages were identical or very similar to each other, and that their genomes were made by a double-stranded DNA molecule of approximately 40–45 Kb. One phage was selected for further characterizations and named φBO1E.

### ΦBO1E genome sequencing and analysis

High-throughput DNA sequencing of the whole phage genome and resolution of the 5′- and the 3′-ends performed by Sanger sequencing showed that the φBO1E genome was 43,865 bp long, with a GC content of 53.8% and two direct repeats of 224 bp located at the 5′- and 3′-ends.

**Figure 1:**
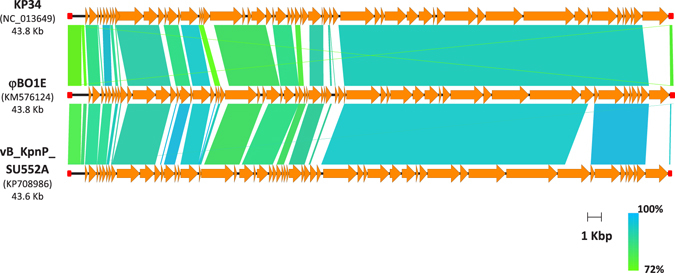
Comparison of the φBO1E genome with KP34, the type phage of the *Kp34virus* genus, and with vB_KpnP_SU552A, the closest homologous deposited in public nucleotide databases. For each phage the corresponding accession numbers and the size in Kb are reported on the left. Long terminal repeats (LTR) are indicated by red squares.

Bioinformatic analysis revealed 59 open reading frames (ORF) (Supplementary Table S1), all encoded on the same DNA strand (Fig. 1). Comparison with phages genomes deposited in the International Nucleotide Sequence Database Collaboration (INSDC) databases revealed that φBO1E is a newly discovered bacteriophage most closely related with the *K. pneumoniae* vB_KpnP_SU552A phage (overall nucleotide identity, 87%), a member of the *Podoviridae* family, *Autographivirinae* subfamily^22^. Similar to vB_KpnP_SU552A, φBO1E encoded a single subunit RNA polymerase, a common characteristic among members of the *Autographivirinae* subfamily, retained the same predicted host and phage promoters as well as a putative rho-independent transcriptional terminator, and was characterized by a lysis module composed by spanin-, holin- and endolysin-encoding genes located next to each other at the 3′-end of the phage genome. Altogether, these findings suggested that φBO1E belongs to the newly proposed genus *Kp34virus*, recently approved by the International Committee on Taxonomy of Viruses^22, 23^.

### Electron microscopy

**Figure 2:**
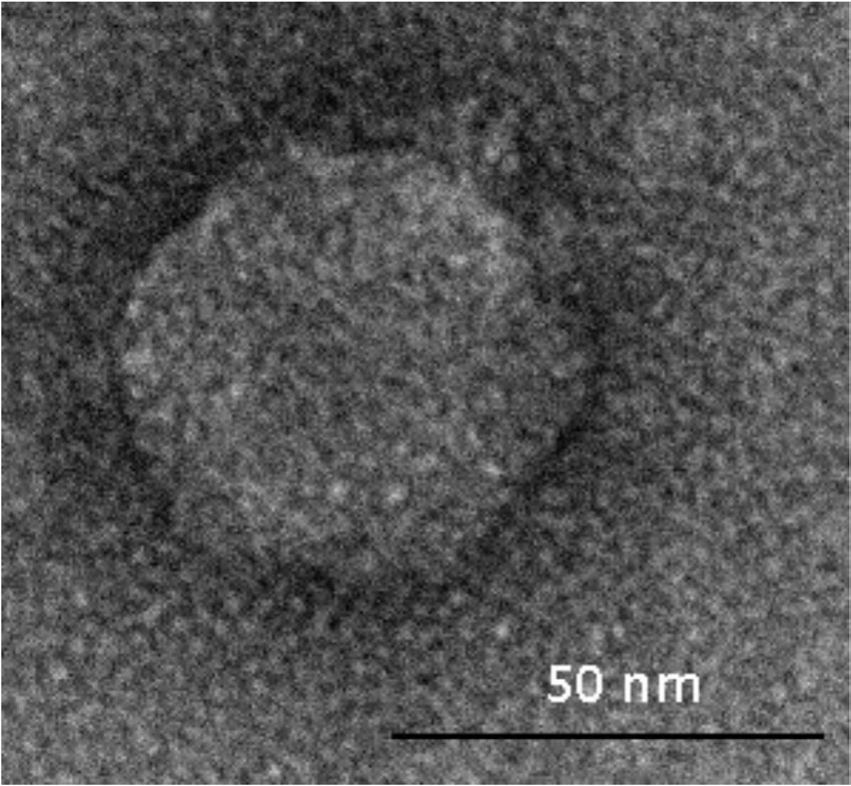
Transmission electron micrograph of phage φBO1E negatively stained with uranyl acetate. The bar indicates 50 nm.

Results of analysis performed with transmission electron microscopy (TEM) showed that φBO1E has morphological features typical of the members of the family *Podoviridae* and order *Caudovirales*, characterized by a head of icosahedral symmetry with a diameter of approximately 50 nm and a short tail of 8–10 nm (Fig. 2). This result was consistent with the estimated size of the genome, considering that *Podoviridae* generally have a genome of about 38–45 Kb.

### Determination of φBO1E bacterial host range

Results from spot tests, carried out with a collection of 82 clinical strains of different clonal lineages and capsular types of *K. pneumoniae* (Supplementary Table S2) demonstrated that φBO1E has a unique lytic specificity towards strains belonging to clade II of CG258 (e.g. ST258, ST512, ST554 and ST1879) and characterized by a CPS_KKBO-4_/*wzi*154-type capsular gene cluster, all of which were lysed (n = 43). Strains having different CPS (n = 39), including those of ST258 clade I (CPS_KK207-2_/*wzi*29-K41-type), were not sensitive to the action of the phage particles (Supplementary Table S2). The ability of φBO1E to exclusively lyse CG258 clade II strains, but not those of CG258 clade I, suggests that the cell structures recognized by the phage are components of the capsular polysaccharide, since the main difference between the two clades is the production of a different type of CPS^13, 14^.

### Sensitivity of φBO1E to temperature and pH

**Figure 3:**
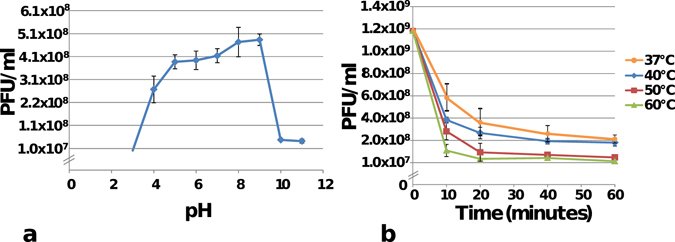
(**a**) Influence of pH towards the infection ability of φBO1E. Phage suspensions were incubated for 1 hour at the different pH values indicated by diamonds. (**b**) Effect of temperature towards the infection ability of φBO1E. Results obtained with different temperatures, and times of incubations are shown. In both cases data are the mean of three independent experiments. Vertical black bars represent one standard deviation.

Results of experiments performed to assess stability of phage particles to temperature and pH demonstrated that φBO1E was stable within a wide range of conditions. In particular, a notable decrease of the infective capacity ( ≥ 2 log) was observed only after incubation at pH < 4 or > 9 (Fig. 3a), or after incubation at a temperature ≥ 50 °C for at least 60 minutes (Fig. 3b). These results demonstrated that φBO1E is more stable to temperature than previously described phages of the *Kp34virus* genus, which after 10 minutes at 60 °C exhibited a remarkable decrease in titer^22^.

### Determination of phage life cycle

Plaques of φBO1E on the indicator strain KKBO-1 showed a clear appearance, with no colonies growing inside the halos, suggesting a lytic nature of the phage. To assess the possibility of lysogenization, bacterial cells were infected for two rounds with φBO1E at increasing multiplicity of infection (MOI), as described in the methods section. The cells which survived this exposure were confirmed to be phage-resistant because PCR screening with primers specific to the φBO1E depolymerase gene yielded no amplicons. Altogether these results demonstrated that cells were not φBO1E lysogens and that the phage is unable to lysogenize its *K. pneumoniae* host.

### One-step growth experiments

**Figure 4:**
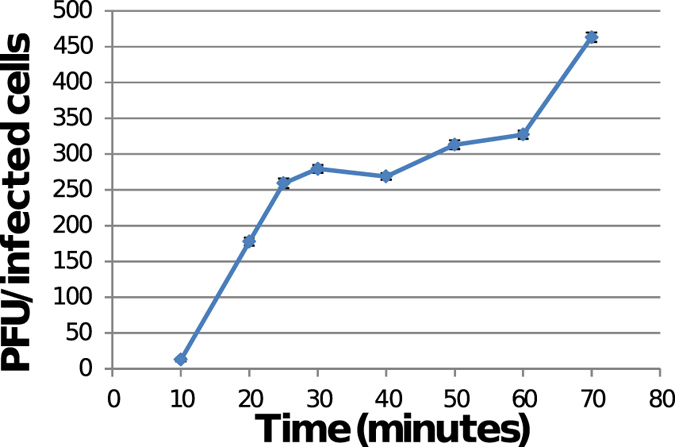
One-step growth curve of bacteriophage φBO1E. Ratios between PFU and the number of infected bacterial cells at different times are shown. Data are the mean of three independent experiments. Vertical black bars represent one standard deviation.

Results of burst-size experiments, reported in Fig. 4, showed that the phage is characterized by a relatively short latency period (approximately 10 minutes). The period of increase of the phage progeny was about 20 minutes before a *plateau* level is reached at about 30 minutes. The computed burst-size was of ≈300 phage particles per infected cell.

### Protection from infection in the *G. mellonella* infection model

To verify the possible *in vivo* efficacy of φBO1E in protecting against infection, the *G. mellonella* wax moth larvae model was employed. Larvae were injected with suspensions of two well characterized KPC-KP strains of the CG258 clade II, KKBO-1 and KP04C62^24, 25^. The former strain is a representative of the major KPC-KP clone circulating in Italy during the early phases of the epidemic^17^, while the latter is a representative of ST512, which emerged subsequently and currently is one of the most prevalent KPC-KP clones circulating in Italy^16^. The latter strain is also colistin resistant and exhibits a hypermucoviscous phenotype^25^.

**Figure 5:**
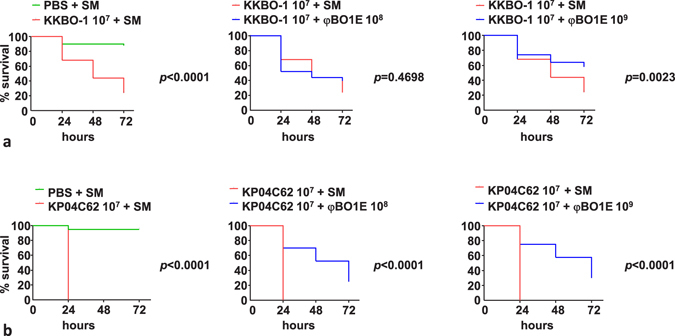
Kaplan–Meier survival curves of *Galleria mellonella* larvae infected with 10^7^ cells of KKBO-1 (**a**) or KP04C62 (**b**) and treated with phage φBO1E at a Multiplicity of Infection (MOI) of 10 or 100 at 30 minutes post-infection. Results obtained with control larval groups with the same amount of bacterial cells but not treated with phage are also shown. Abbreviations are as follow: PBS, phosphate buffered saline; SM, Salt Medium.

In the *G. mellonella* model, both strains exhibited a very similar lethal dose 50% (LD_50_) (KKBO-1, log LD_50_: 6.02 ± 0.09 CFU; KP04C62, log LD_50_: 6.1 ± 0.05 CFU)^25, 26^. Experiments of phage protection, performed with a tenfold LD_50_ bacterial challenge (10^7^ CFU), revealed that treatment with φBO1E was overall capable of protecting *G. mellonella* from lethal infection (Fig. 5), even if with differences between the two strains. In fact, with KKBO-1 differences in mortality rates obtained with MOI 10 was not significant (difference of 14% at 72 h, *p* = 0.4698), while a significant protection was observed using an MOI of 100 (difference of 34% at 72 h, *p* = 0.0023) (Fig. 5a). Conversely, with KP04C62 significant protection was observed with both MOIs (Fig. 5b; difference at 72 h of 43%, *p* < 0.0001 and 35%, *p* < 0.0001 with MOI 10 and 100, respectively). The reasons for this difference remain unknown. Possible explanations could be: i) the effect of the phage is easier to observe with KP04C62 because larvae die faster or ii) a reduction of the capsule-mediated virulence of the KP04C62 strain connects to the activity of the phage virion-associated depolymerase.

## Discussion

The advent of antibiotic resistance crisis, with the global dissemination of bacterial pathogens which are resistant to most of the available therapeutic options, has renewed the interest for alternatives to antimicrobial compounds. The use of lytic bacteriophages as antibacterial agents had been considered in the early 1900s^27^, and then largely abandoned in favor of antibiotics. Recently however phages, or components thereof, have been reconsidered with interest as potential antibacterial agents^28–30^. Advantages of bacteriophages and their components include activity against antibiotic resistant bacteria and targeting of a very narrow-spectrum of infecting pathogens, with minimal impact on the resident microbiota.

The φBO1E phage described in this work is a newly discovered lytic bacteriophage of the *Podoviridae* family able to specifically lyse CG258 KPC-KP of clade II. To the best of our knowledge this report constitutes the first example of a lytic phage that specifically targets this lineage. Indeed, other bacteriophages against KPC-KP of the *Podoviridae* and *Myoviridae* families have been previously described, but their target specificity was not investigated^31, 32^.

The phage described in this work is characterized by a number of favorable features, such as a notable stability to a wide range of pH and temperatures, a strict lytic nature, and the absence in its genome of genes encoding potential toxins. These features provide a strong rationale for the possible use of this phage or proteins thereof against KPC-KP strains belonging to the CG258 clade II lineage.

Indeed, KPC-KP of CG258 were shown to be among the major drivers for the KPC-KP pandemic, with a predominance of clade II strains^14, 16, 20^. Under these circumstances, the lytic bacteriophage described in this work could be of relevant interest as a tool for decolonization of mucosal surfaces or even for the treatment of some infections and it could be used, alone or in phage-cocktails, to treat patients according to protocols of personalized medicine^33, 34^.

The efficacy of φBO1E in reducing mortality of *G. mellonella* larvae infected by KPC-KP of CG258 clade II supports a potential utility of this phage for the above mentioned applications, although further experiments using different MOIs, times of phage administration and animal hosts (e.g. mouse model) will be necessary to confirm this hypothesis.

The specificity of φBO1E for this clade suggests that the phage recognizes specific structures exposed on the capsular polysaccharide, given the fact that the major difference between the ST258 clades is the expression of distinct CPS and that it was recently demonstrated that strains of both CG258 clades express an identical lipopolysaccharide, termed D-galactan III^35^. The characterization of the molecular targets recognized by φBO1E will be of paramount importance both to elucidate the infection process of the phage and also to understand the mechanisms leading to resistance to infection, and will be the subject of future investigations. In addition, it would also be interesting to test phages related to φBO1E for their host-spectrum against CG258 strains.

## Materials and Methods

### Bacterial strains

The clinical isolate KKBO-1, a previously characterized KPC-KP strain of ST258 clade II^14, 24^, was used as host for phage isolation and propagation. The same strain, along with the clinical isolate KP04C62, a KPC-KP strain of ST512 clade II that was also colistin-resistant and expressed an RmpA-independent hypermucoviscous phenotype, were used in the *G. mellonella* infection experiments.

### Phage isolation

Bacteriophage φBO1E was recovered from untreated hospital wastewaters by using the top-agar (lysogeny broth (LB) with 0.7% agar (Oxoid, Hampshire, UK)) overlay method and KKBO-1 as indicator strain, as previously described^23^ with some modifications. Briefly, 40 ml of wastewaters were centrifuged at 3,850 × *g* at 25 °C for 10 minutes. The supernatant was then filtered sequentially through 0.45 µm and 0.22 µm filters and diluted 1:100 in sterile ddH_2_O. An aliquot of 100 µl of this dilution was then used in the top-agar overlay experiment. Three rounds of infection and picking of isolated plaques were performed to obtain pure bacteriophage suspensions.

### Determination of the host spectrum

The ability of φBO1E to target different clones of *K. pneumoniae* was assessed by the spot test technique, using 82 previously characterized KPC-KP clinical strains (Supplementary Table S2), as previously described^36^.

### Large-scale production of bacteriophage suspensions

Amplification of bacteriophage φBO1E was performed as follows. An overnight (O/N, corresponding to 12–18 hours) culture of KKBO-1 in LB broth, concentrated to 1/10 of its initial volume in salt medium (SM) buffer^37^ (final volume 200 µl), was mixed with 100 µl of a bacteriophage suspension with a titer of 10^8^ PFU/ml. After incubation for 30 minutes at 37 °C, 5 ml of molten top-agar were added and the obtained mixture was poured onto an LB agar (LBA) plate. Ten LBA plates were prepared and, following an O/N incubation at 37 °C, 4 ml of SM buffer were added to each plate. Subsequently, after 2 hours of incubation at room temperature (RT) with gentle shaking, the top-agar layer together with SM buffer were recovered using a scraper. The obtained mixture was finally centrifuged, and the supernatant was decanted, filtered through 0.22 µm filter and stored at 4 °C.

### Electron microscopy

A bacteriophage suspension containing ≈10^12^ PFU/ml, obtained by centrifugation at 25,000 × *g* for 60 minutes of 10 ml of the mixture from large-scale production of the phage, followed by resuspension of the pellet in 100 μl of SM buffer (pH = 7.5), was used for electron microscopy analysis. Preparations of bacteriophages particles were processed by standard negative stain^38^, and observed by a FEI Tecnai 12 (FEI, Eindhoven, The Netherlands) TEM fitted with an Osis Morada 2X4 K CCD camera (Olympus, Shinjuku, Tokyo, Japan). In detail, 10 μl of the concentrated phage suspension were let to adsorb on a carbon-coated matrix and then stained with 2% uranyl acetate for 15 seconds. The grid was subsequently washed 2 times with ddH_2_O, air dried and imaged by TEM.

### Sensitivity to physical agents

Stability of φBO1E at different temperatures was determined by diluting phage particles to a final concentration of ≈10^9^ PFU/ml in a final volume of 1 ml of SM buffer. The aliquots were incubated at 25 °C (control), 37 °C, 40 °C, 50 °C and 60 °C, for 10, 20, 40 and 60 minutes. After incubation, the phage suspensions were titrated. Stability of φBO1E at different pH values was determined by diluting phage particles to a final concentration of 5 × 10^8^ PFU/ml in SM buffer previously brought to different pH values using 1 M NaOH or 1 M HCl, to create a pH range from 3 to 11 with intervals of 1 unit. Phage suspensions were incubated for 60 minutes at 25 °C and then titrated. Assays for the determination of the stability to temperature and pH were made in triplicate and the reported values are the mean of the observations ± standard deviation.

### Determination of burst-size

The burst-size of the φBO1E phage, representing the average number of phages released by each bacterium after the lysis, was determined as follows. The indicator strain KKBO-1 was inoculated in 5 ml of LB and the inoculum was grown under aerobic conditions at 37 °C until the exponential phase of growth (OD_600_ = 0.3–0.4). An aliquot of 1 ml of the inoculum was then centrifuged at 13,000 × *g* for 5 minutes and the pellet was resuspended in 1 ml of SM buffer. To 0.9 ml of this suspension 0.1 ml of the phage lysate at a concentration of 1 × 10^7^ PFU/ml was added, in order to achieve an MOI of 0.01. The mixture was incubated for 10 minutes in a water-bath at 37 °C with shaking, centrifuged at 13,000 × *g* for 4 minutes at RT and resuspended in 1 ml of SM buffer to remove any non-adsorbed phages. This preparation was then diluted 1:10,000 in LB medium (10 ml) and incubated in a water-bath at 37 °C. Aliquots of 0.1 ml were sampled after 10, 20, 25, 30, 40, 50, 60 and 70 minutes. Each aliquot was added to 4.5 ml of top agar, mixed, poured onto an LBA plate and incubated O/N at 37 °C. The latency period was defined as the time between infection (excluding the 15 minutes of pretreatment) and the shortest incubation time allowing the production of phages^39^. The burst-size was calculated as the ratio between the number of phage particles released at the *plateau* level and the initial number of infected bacterial cells. Experiment was performed three times and the reported values are the mean of the observations ± standard deviation.

### Lysogeny verification

To verify the tentative temperate nature of the isolated phage, exponentially growing cultures of KKBO-1 in LB broth were infected with phages at an MOI of ~1 and incubated at 37 °C with shaking at 200 rpm for 24 hours. The infected culture was then diluted 1:100 in LB broth and phage stock suspension was added at an MOI > 1 × 10^4^. After 1 hour of incubation at 37 °C with shaking at 200 rpm, 0.1 ml were plated on LBA and incubated O/N at 37 °C, thus allowing the growth of presumptive φBO1E lysogen cells. Nine single colonies were picked and isolated in pure cultures. Resistance against φBO1E infection was tested by spotting 5 µl of the phage stock suspension (corresponding to 5 × 10^7^ PFU) on LB agar plates overlaid with 4.5 ml top-agar containing the presumptive φBO1E lysogens. Obtained colonies were re-identified at the species level, investigated by multiplex PCR assay for *cps* genotyping^14^ and subjected to PCR amplification using primers targeting the φBO1E depolymerase gene (CDS59_F 5′-ATGAATTTAGTAAAAGCAAAGTATCCG-3′ and CDS59_R 5′-CTAGAAAGCTGCCTGGGTATC-3′).

### DNA isolation and restriction endonuclease analysis

Extraction of the phage genomic DNA was performed using the Wizard^®^ DNA Clean-Up System (Promega, Madison, WI, USA), following manufacturer recommendations. The exact determination of phage genomic DNA concentrations was carried out using a NanoDrop apparatus (Nanodrop Technologies Inc., Wilmington, USA), following the instructions provided by the manufacturer. Phage DNA preparations were digested with HindIII and BamHI restriction endonucleases (New-England Biolabs, MA, USA) for 8–10 hours. Restriction fragments were separated by electrophoresis in 0.75% agarose gel, stained with ethidium bromide and visualized under ultra-violet light to roughly estimate the genome size.

### Genome sequencing

The genome of φBO1E was sequenced using a MiSeq instrument (Illumina Inc., San Diego, CA, USA) and a paired-ends approach (2 × 250 bp) with the kit Illumina Nextera™. Characterization of the ends of the phage genome was performed by direct Sanger sequencing of bacteriophage DNA, using the primers BO1E_2 F (5′-TTGACTACGTCGGGATAGGC-3′) and BO1E_1 R (5′-AGCACTAGCGATAGCCAGTG-3′), as described elsewhere^40^.

### Bioinformatic analysis

Reads obtained by high-throughput sequencing were trimmed usingDynamicTrim.plscript with a h value of 30 and assembled using the ABySS software^41, 42^. A single contig of 43.8 Kb was thus obtained and annotated using the RAST web-service^43^. Automatic annotation was manually reviewed by BLASTP analysis against Refseq proteins deposited in INSDC databases. Conserved protein domains were searched for by using the web based CD-Search tool^44^, and results were filtered to remove non-specific hits. Putative host and phage promoters, and rho-independent transcriptional regulators were annotated by comparison with the closest homologs deposited in the INSDC databases: KP34 (Accession number: NC_013649), vB_KpnP_SU503 (Accession number: KP708985), vB_KpnP_SU552A (Accession number: KP708986), NTUH-K2044-K1-1 (Accession number: AB716666) and F19 (Accession number: KF765493).

### *G. mellonella* phage therapy assay

Larvae of *G. mellonella* were obtained from Sa.gi.p (Sa.gi.p, Ravenna, Italy) and used after one O/N incubation at 14 °C. Larvae were inspected to select candidates weighing approximately 450–600 mg for the phage therapy assay. *G. mellonella* larvae were surface-disinfected with a cotton swab dipped in 70% ethanol (Sigma-Aldrich) and injected with 10 μl of inoculum containing 10^7^ cells of *K. pneumoniae* KKBO-1 or *K. pneumoniae* KP04C62 into the larval haemolymph behind the last proleg, by using a 30-gauge syringe (Hamilton, Reno, NV). Bacterial suspensions were prepared in 10 mM phosphate buffered saline (PBS) pH 6.5. At 30 minutes post-infection, a group of larvae was injected at the same site, but on the opposite side to the bacterial injection, with 10 μl of a φBO1E preparation at a concentration to obtain an MOI of 10 or 100. A total of 10 larvae was used for each condition. Positive (larvae infected with *K. pneumoniae* KKBO-1 or KP04C62 and treated with SM buffer) and two negative control groups (one group injected with SM buffer only and one group injected with phage suspension only) were also included. Larvae were placed into Petri dishes and incubated at 35 ± 2 °C in the dark, in humidified atmosphere, with food, and daily examined for pigmentation and mobility. Time of death was recorded at 24, 48 and 72 hours. For each experiment, the injected inoculum was checked by plating serial dilutions and enumerating colonies after 12–16 hours of incubation at 37 °C. Five independent experiments were performed for each different bacterial inoculum/phage titer combination. Data from independent experiments were pooled and the protection of larvae from death by φBO1E was assessed by log-rank (Mantel-Cox) test. *p* values < 0.05 were considered statistically significant. Statistical analyses were performed using GraphPad Prism software (GraphPad Software, Inc., La Jolla, USA).

## Electronic supplementary material

Supplementary Table S1 and S2
