# Supplementary material for: φBO1E, a newly discovered lytic bacteriophage targeting carbapenemase-producing Klebsiella pneumoniae of the pandemic Clonal Group 258 clade II lineage
Source: Sci Rep. 2017 Jun 1;7:2614. doi: 10.1038/s41598-017-02788-9 (PMC5453958; doi:10.1038/s41598-017-02788-9)
Supplement: Supplementary file 1 — Supplementary Table S1 and S2 [file 41598_2017_2788_MOESM1_ESM.pdf]

Supplementary information

φBO1E, a newly discovered lytic bacteriophage targeting carbapenemase-producing *Klebsiella pneumoniae* of the pandemic Clonal Group 258 clade II lineage

Marco Maria D'Andrea[1,2*], Pasquale Marmo[3], Lucia Henrici De Angelis[1], Mattia Palmieri[1], Nagaia Ciacci[1,3], Gustavo Di Lallo[3], Elisa Demattè[1], Elisa Vannuccini[4], Pietro Lupetti[4], Gian Maria Rossolini[1,2,5] and Maria Cristina Thaller[3]

[1] Department of Medical Biotechnologies, University of Siena, Siena, Italy
[2] Department of Experimental and Clinical Medicine, University of Florence, Florence, Italy
[3] Department of Biology, University of Rome "Tor Vergata", Rome, Italy
[4] Department of Life Sciences, University of Siena, Siena, Italy
[5] Clinical Microbiology and Virology Unit, Florence Careggi University Hospital, Florence, Italy

Supplementary Table S1. General features of genes encoded in the genome of phage φBO1E. For each predicted ORF the start and the stop positions, the predicted function of the encoded protein and its closest homolog found in INSDC databases, together with the degree of identity and coverage and the phage from which the ORF derived, are reported. Last column shows the conserved domains detected in some proteins, the associated accession numbers and the region in which a particular domain was found.

CDS No	Start	Stop	Predicted Function	Closest Protein Homolog	Query cov (%)	ID (%)	Phage	Conserved domains (from-to Accession (short name))
1	1507	1722	hypothetical protein	YP_009199885.1	100	87	vB_KpnP_SU503	
2	1792	2367	Phage protein	YP_009204791.1	100	95	vB_KpnP_SU552A	
3	2438	2659	hypothetical protein	YP_009199887.1	97	77	vB_KpnP_SU503	
4	2718	2933	hypothetical protein	YP_003517733.1	100	93	KP34	
5	2926	3189	hypothetical protein	YP_003347656.1	100	97	KP34	
6	3205	3444	hypothetical protein	YP_009188748.1	97	87	vB_KpnP_KpV41	4-66 cl05604 (DUF987 superfamily)
7	3444	3626	hypothetical protein	YP_009188749.1	98	92	vB_KpnP_KpV41	
8	3623	3835	hypothetical protein	YP_009188319	100	71	vB_Kp2	

9	3823	4311	Phage-associated homing endonuclease	YP_009224671.1	90	51	Peat1	64-98 pfam13392 (HNH_3)<br>64-98 cl00083 (HNHc superfamily)
10	4308	4652	hypothetical protein	YP_009188751.1	100	87	vB_KpnP_KpV41	
11	4696	6369	Phage protein	YP_009006029.2	99	92	F19	
12	6369	7424	Phage protein	YP_009098356.1	100	97	NTUH-K2044-K1-1	106-244 cl14876 (Zinc_peptidase_like superfamily)
13	7427	7891	hypothetical protein	YP_009098357.1	98	44	NTUH-K2044-K1-1	2-43 cl17172 (ADH_N superfamily)
14	7884	8090	hypothetical protein	YP_009006032.1	100	49	F19	
15	8090	8875	DNA primase/helicase, phage-associated	YP_009188325.1	99	97	vB_Kp2	164-251 cl00718 (TOPRIM superfamily)<br>1-260 PHA02031 (PHA02031)<br>189-261 COG0358 (DnaG)
16	8872	9045	hypothetical protein	-	-	-	no significant similarity	
17	9046	10326	DNA helicase, phage-associated	YP_009188758.1	99	99	vB_KpnP_KpV41	175-345 pfam13481 (AAA_25)<br>175-345 cl21455 (P-loop_NTPase superfamily)<br>95-342 TIGR03600 (phage_DnaB)<br>150-230 COG0305 (DnaB)
18	10319	10645	hypothetical protein	YP_009188328.1	91	94	vB_Kp2	

19	10697	10918	hypothetical protein	YP_009188329.1	94	96	vB_Kp2	
20	10876	11031	hypothetical protein	YP_009188759.1	100	92	vB_KpnP_KpV41	
21	11028	11384	hypothetical protein	YP_009199901.1	100	57	vB_KpnP_SU503	66-104 cl10231 (PHA00527 superfamily)
22	11374	13719	DNA polymerase I (EC 2.7.7.7), phage-associated	YP_009188330.1	100	88	vB_Kp2	374-745 cl02626 (DNA_pol_A superfamily)<br>81-144 cl10012 (DnaQ_like_exo superfamily)<br>444-779 PRK14975 (PRK14975)<br>350-745 COG0749 (PolA)<br>379-776 TIGR00593 (PolA)<br>379-525 PRK05755 (PRK05755)
23	13716	13937	hypothetical protein	YP_009199904.1	100	95	vB_KpnP_SU503	
24	14099	14377	hypothetical protein	YP_003347673.1	100	98	KP34	
25	14377	15372	Phage protein, putative phosphoesterase	YP_009188763.1	100	97	vB_KpnP_KpV41	99-260 pfam00149 (Metallophos)<br>99-260 cl13995 (MPP_superfamily superfamily)
26	15392	15580	hypothetical protein	YP_003347676.1	56	89	KP34	6-31 cl22805 (small_Trp_rich superfamily)
27	15636	16406	Phage protein, large tegument protein	YP_009188336.1	100	97	vB_Kp2	1-228 PHA02030 (PHA02030)
28	16460	16714	hypothetical protein	YP_009188337.1	100	98	vB_Kp2	

29	16718	17092	hypothetical protein	YP_009199910.1	98	94	vB_KpnP_SU503	
30	17092	17253	hypothetical protein	YP_009204815.1	100	70	vB_KpnP_SU552A	
31	17257	17418	hypothetical protein	YP_009199911.1	100	62	vB_KpnP_SU503	
32	17418	18386	Phage protein, putative 5'-3' exonuclease	YP_009006045.1	100	95	F19	
33	18343	18543	hypothetical protein	YP_009204819.1	100	91	vB_KpnP_SU552A	
34	18537	18680	putative homing endonuclease	YP_009102767.1	76	49	phD2B	
35	18677	19099	Phage DNA endonuclease VII	YP_009188344.1	100	68	vB_Kp2	16-102 cl03794 (Endonuclease_7 superfamily)<br>36-72 cl03772 (Peptidase_S29 superfamily)<br>5-101 PHA02565 (49)
36	19327	19563	Phage protein	YP_009199915.1	97	99	vB_KpnP_SU503	5-66 pfam11753 (DUF3310)<br>5-66 cl13237 (DUF3310 superfamily)
37	19586	20005	Phage-associated homing endonuclease	YP_007392463.1	87	46	φTE	41-85 pfam13392 (HNH_3)<br>41-85 cl00083 (HNHc superfamily)<br>44-123 PHA00280 (PHA00280)
38	19986	20108	hypothetical protein	YP_009188346.1	92	92	vB_Kp2	
39	20147	22615	Phage DNA-dependent RNA polymerase (EC 2.7.7.6)	YP_009188347.1	100	98	vB_Kp2	431-822 pfam00940 (RNA_pol)<br>431-822 cl20211 (RNA_pol superfamily)<br>17-302 pfam14700 (RPOL_N)

								17-302 cl20638 (RPOL_N superfamily)<br>11-822 PHA00452 (PHA00452)<br>3-821 COG5108 (RPO41)
40	22639	23079	hypothetical protein	YP_009006053.2	100	99	F19	
41	23076	23339	hypothetical protein	YP_003347631.1	100	100	KP34	
42	23349	24944	Phage head-tail (collar) connector protein	YP_003347633.2	100	98	KP34	24-488 cl19541 (Head-tail_con superfamily)
43	24959	25801	Phage scaffolding protein	YP_009199921.1	100	98	vB_KpnP_SU503	12-280 PHA01929 (PHA01929)
44	25827	26846	Phage capsid and scaffold	YP_009204828.1	100	99	vB_KpnP_SU552A	4-338 cl21666 (PHA02004 superfamily)
45	26858	27040	hypothetical protein	YP_003347637.1	100	100	KP34	
46	27129	27689	putative phage tail fibers,<br>tail tubular protein A	YP_009188354.1	100	97	vB_Kp2	1-173 cl10205 (PHA00428 superfamily)
47	27699	30071	putative phage tail fibers,<br>tail tubular protein B	YP_009199925.1	98	98	vB_KpnP_SU503	343-513 cl20016 (PHA00661 superfamily)
48	30074	30676	putative internal virion protein B	YP_009098376.1	100	80	NTUH-K2044-K1-1	
49	30692	33376	Phage protein	YP_009188357.1	100	98	vB_Kp2	

50	33427	37125	Methyl-accepting chemotaxis protein I (serine chemoreceptor protein), putative internal core protein	YP_009199928.1	100	99	vB_KpnP_SU503	1-1231 cl20117 (PHA03413 superfamily)<br>35-1203 cl20118 (PHA03414 superfamily)<br>585-1178 cl20119 (PHA03415 superfamily)
51	37127	38083	Phage tail fibers	YP_009199929.1	100	89	vB_KpnP_SU503	1-138 cl04321 (Phage_T7_tail superfamily)<br>14-174 PHA00430 (PHA00430)
52	38095	38397	Phage protein, putative DNA maturase A	YP_003347644.1	100	94	KP34	1-98 PHA02046 (PHA02046)<br>1-98 cl10354 (PHA02046 superfamily)<br>2-72 PHA03334 (PHA03334)
53	38397	40253	Phage DNA packaging, putative DNA maturase B	YP_009204837.1	100	99	vB_KpnP_SU552A	55-113 smart00487 (DEXDc)
54	40253	40627	hypothetical protein	YP_003347646.1	100	99	KP34	
55	40639	40821	hypothetical protein	YP_003347647.1	83	92	KP34	
56	40821	41225	Phage protein, putative spanin	YP_009188794.1	100	99	vB_KpnP_KpV41	
57	41218	41469	putative holin / transmembrane protein	YP_009006072.1	100	100	F19	
58	41498	42064	Putative phage lysozyme (endolysin)	YP_009204842.1	96	95	vB_KpnP_SU552A	14-117 cl00222 (lysozyme_like superfamily)
59	42061	43545	hypothetical protein	KGT08080.1	12	41	NV63_18575	16-61 cl19188 (Pectate_lyase_3 superfamily)<br>23-58 cl13628 (End_N_terminal superfamily)<br>15-57 PLN02218 (PLN02218)<br>4-45 COG5434 (Pgu1)

Supplementary Table S2. Results of the test for determination of the host spectrum of φBO1E. For each combination of sequence type/*wzi* variant, the number of tested strains, the deduced capsular type and the result of the observed lysis are reported (-=not lysed;+=lysed; ND=Not deducible).

No of Strains[a]	Sequence type	Capsular genotype*	Deduced capsular serotype	lysis
1	ST1879	*wzi*154	KKBO-4	+
19	ST258	*wzi*154	KKBO-4	+
22	ST512	*wzi*154	KKBO-4	+
1	ST554	*wzi*154	KKBO-4	+
9	ST258	*wzi*29	K41(KK-207)	-
4	ST101	*wzi*137	K17	-
1	ST11	*wzi*75	ND	-
1	ST11	*wzi*2	K2	-
1	ST14	*wzi*2	K2	-
4	ST147	*wzi*64	K14/K64	-
5	ST15	*wzi*24	K24	-
1	ST160	*wzi*149	K62	-
1	ST1633	*wzi*137	K17	-
1	ST17	*wzi*64	K14/K64	-
1	ST17	*wzi*141	ND	-
1	ST208	*wzi*35	K35	-
1	ST307	*wzi*174	ND	-
1	ST307	*wzi*173	ND	-
1	ST340	*wzi*50	K15/K17/K50/K51/K52	-
1	ST37	*wzi*96	K38	-
1	ST383	*wzi*134-K30(6 mismatches)	ND	-
1	ST45	*wzi*101	ND	-
1	ST641	*wzi*38	K38	-
2	ST945	*wzi*87 (1 mismatch)	K59	-

[a] tested strains were from different nations, hospitals, samples and patients and were mostly epidemiological unrelated

* capsular genotyping was performed using the *wzi* method described by Brisse and colleagues (Brisse, S. *et al. wzi* gene sequencing, a rapid method for determination of capsular type for *Klebsiella* strains. *J. Clin. Microbiol.* **51**, 4073-4078 (2013))
